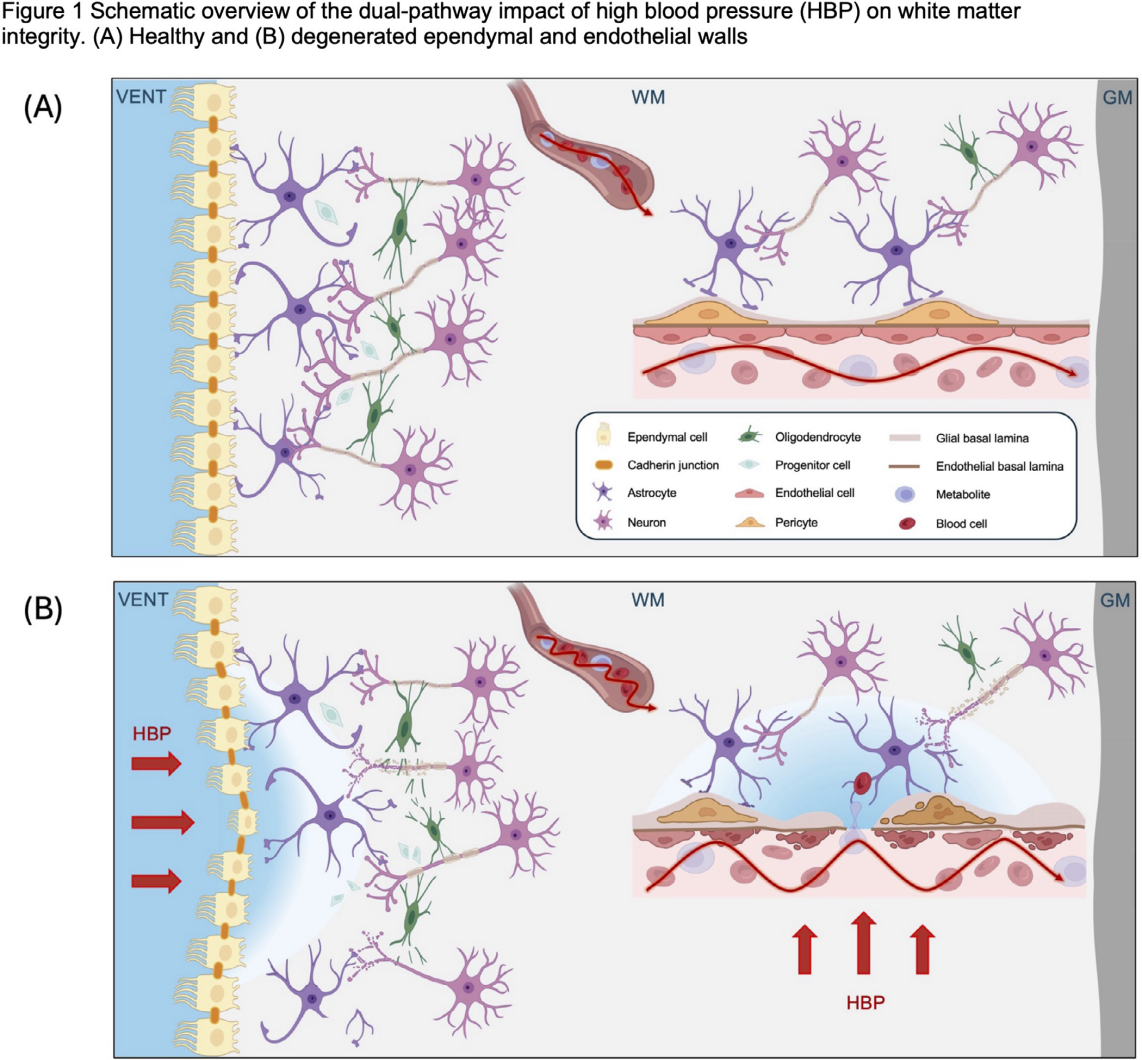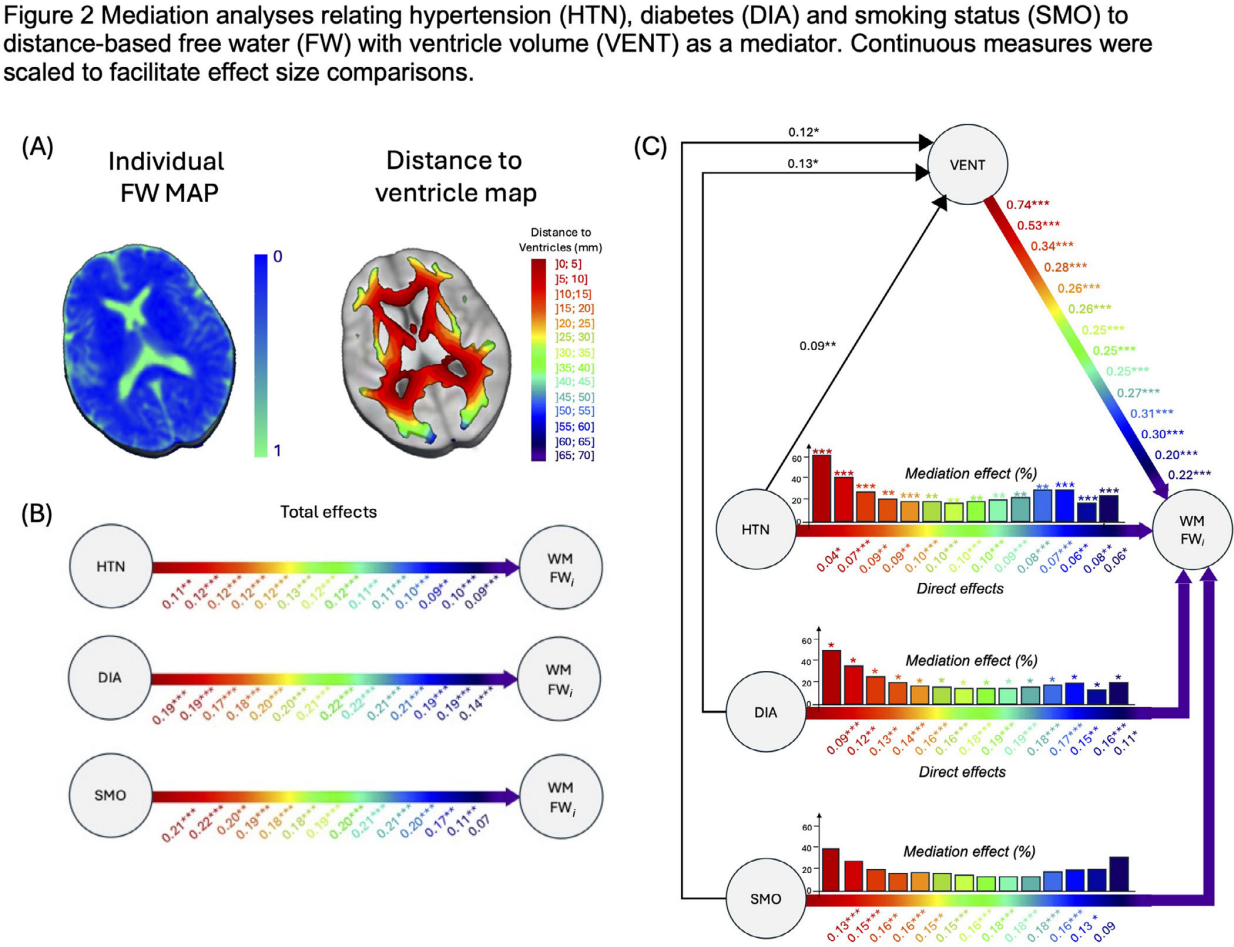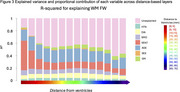# A dual‐pathway hypothesis to explain free water white matter spatial distribution related to vascular risk factors

**DOI:** 10.1002/alz70856_106149

**Published:** 2026-01-07

**Authors:** Pauline Maillard, Alexa S Beiser, Claudia L Satizabal, Johannes Weickenmeier, Sudha Seshadri, Charles Decarli

**Affiliations:** ^1^ Department of Neurology and Center for Neuroscience, University of California, Davis, Davis, CA, USA; ^2^ Department of Biostatistics, Boston University School of Public Health, Boston, MA, USA; ^3^ University of Texas Health San Antonio, San Antonio, TX, USA; ^4^ University of Oxford, Oxford, Oxfordshire, United Kingdom; ^5^ Department of Neurology & Imaging of Dementia and Aging Laboratory, University of California Davis, Sacramento, CA, USA

## Abstract

**Background:**

DTI‐derived free water (FW) is a marker for early cerebral white matter (WM) injury, occurring before white matter hyperintensities (WMH). Both increased FW and WMH are associated with aging, hypertension, and raised arterial stiffness. Enlarged ventricles and arteriosclerosis are common features associated with these processes. Recent work^1^ finds enlarged ventricular volume is associated with periventricular WMH and hypothesizes ependymal leakage as the pathophysiological initiator. We have similarly posited that arterial stiffness leads to endothelial leakage in cerebral WM small vessels^2^. The goal of the present study is to propose a biomechanical hypothesis explaining how high blood pressure promotes WM injury through a dual‐pathway mechanism (Figure 1).

**Method:**

Our study includes 3303 Framingham Heart Study participants (age range: 26‐95; 52% women) with imaging data. WM FW, ventricular (VENT), gray matter (GM) and total cranial volumes (TCV) were derived using methods previously described. VENT and GM volumes were regressed against TCV. For each participant, mean FW measures were computed across 14, 5mm‐spaced WM layers, starting outward from the ventricular surface to the cortex (Figure 2A). We used mediation analyses to investigate the effect of hypertension (HTN), diabetes (DIA) and smoking status (SMO), on distance‐based FW, including VENT as a mediator, adjusting for age, sex and GM.

**Result:**

Total effects of HTN, DIA and SMO on distance‐based FW were significant at all locations (Figure 2B), whereas, VENT had a gradient effect (Figure 2C). VENT differentially mediated the effect of HTN according to distance (*p*‐values<0.01), having least mediation in central WM. Mediation effects were less for DIA (*p*‐values<0.05) and not significant for SMO. VENT was found to have a strong, gradual descending effect on distance‐based FW, suggesting a propagation effect, whereas the combination of HTN and DIA made maximal contribution to variance at central locations (Figure 3).

**Conclusion:**

Spatial analysis of increased FW in response to hypertension and diabetes finds strongest mediation effect by VENT at proximal and explained greatest variance in middle WM locations. We hypothesize that ventricular enlargement and small vessel injury associated with vascular risk factors additively contribute to increased FW in cerebral WM.

1. Cacoilo et al. Brain Multiphysics 2023

2. Maillard et al. Stroke 2017